# A novel maximum likelihood based probabilistic behavioral data fusion algorithm for modeling residential energy consumption

**DOI:** 10.1371/journal.pone.0309509

**Published:** 2024-11-04

**Authors:** Tanmoy Bhowmik, Naveen Chandra Iraganaboina, Naveen Eluru

**Affiliations:** 1 Department of Civil and Environmental Engineering, Portland State University, Portland, OR, United States of America; 2 Department of Civil, Environmental & Construction Engineering, University of Central Florida, Orlando, FL, United States of America; Universita degli Studi del Molise, ITALY

## Abstract

The current research effort is focused on improving the effective use of the multiple disparate sources of data available by proposing a novel maximum likelihood based probabilistic data fusion approach for modeling residential energy consumption. To demonstrate our data fusion algorithm, we consider energy usage by fuel type variables (for electricity and natural gas) in residential dwellings as our dependent variable of interest, drawn from residential energy consumption survey (RECS) data. The national household travel survey (NHTS) dataset was considered to incorporate additional variables that are not available in the RECS data. With a focus on improving the model for the residential energy use by fuel type, our proposed research provides a probabilistic mechanism for appropriately fusing records from the NHTS data with the RECS data. Specifically, instead of strictly matching records with only common attributes, we propose a flexible differential weighting method (probabilistic) based on attribute similarity (or dissimilarity) across the common attributes for the two datasets. The fused dataset is employed to develop an updated model of residential energy use with additional independent variables contributed from the NHTS dataset. The newly estimated energy use model is compared with models estimated RECS data exclusively to see if there is any improvement offered by the newly fused variables. In our analysis, the model fit measures provide strong evidence for model improvement via fusion as well as weighted contribution estimation, thus highlighting the applicability of our proposed fusion algorithm. The analysis is further augmented through a validation exercise that provides evidence that the proposed algorithm offers enhanced explanatory power and predictive capability for the modeling energy use. Our proposed data fusion approach can be widely applied in various sectors including the use of location-based smartphone data to analyze mobility and ridehailing patterns that are likely to influence energy consumption with increasing electric vehicle (EV) adoption.

## 1 Introduction

### 1.1 Background

The United States of America is the second largest consumer of energy with only 4.3% of the world population [1,2]. The energy consumption in the US can be mainly attributed to following sectors: residential use (21%), commercial use (18%), transportation use (29%) and industrial use (32%) [3,4]. Given how individual mobility and activity participation influences energy use, it is not surprising that energy consumption in residential, commercial and transport sectors is intertwined. For instance, households that pursue longer commutes are likely to expend larger energy for transportation and are likely to expend lesser energy at their residence. Similarly, individuals working longer hours at office would contribute to increased energy consumption at commercial buildings and reduced energy use (at least from one individual) in the residence. The intricate relationship among these three sectors became prominent with the ongoing COVID-19 pandemic. Residential energy use increased by 8% during COVID-19 lockdown and/or mobility restrictions (between April to August 2020), while commercial and transportation related energy usage decreased 8% and 21%, respectively [5,6].

With the growing adoption of electric vehicles (EVs), the intricate relationship between energy consumption across sectors will be further strengthened [7,8]. The uptake of EVs and the potential energy source diversification (such as solar and wind energy) would result in a transformation of energy consumption and distribution patterns across the world [8]. The demand for charging the electrical vehicles at home, work and other potential locations is also likely to influence the spatio-temporal nature of the existing electricity demand. It is possible that the current demand on the grid could be rapidly altered with higher residential and commercial demand. There is a growing need for the development of modeling frameworks that provide insights on energy use and potential future energy demand evolution. A major bottleneck for model framework development is the unavailability of “perfect” data.

Recent technological advances and their adoption including sensing technology, smart energy sensors, connected and autonomous vehicles, shared mobility (bike sharing, scooter sharing and transportation network companies), naturalistic driving studies, and location-based smartphone data have resulted in large volumes of data being collected. This data explosion has shifted research challenges in multiple fields from modeling with limited data to developing modeling approaches that support effective utilization of the abundant data. The current research effort is focused on improving effective use of the multiple disparate sources of data available for energy use modeling by proposing a novel maximum likelihood based probabilistic data fusion approach.

Data fusion algorithms refers to the techniques of integrating two or more distinct data sources into a fused data that offers enriched information (additional explanatory variables) compared to the individual data sources [9]. The algorithms can be simple merging efforts across multiple datasets. Let us consider the compilation of a typical residential energy demand dataset. Utility companies compile energy use data using a smart energy sensor system with detailed information on energy demand in continuous time while also compiling residential unit characteristics (such as floor area and the number of bedrooms). The data also has unique information in terms of the residential unit location. Employing the location information, the dataset can be augmented with a Weather and Geographic Information System (GIS) file that provides location specific characteristics such as temperature and precipitation. The merging of data described here is a simple, deterministic fusion. Given the location, using GIS and appropriate weather data, the analyst can query or cross-reference for weather characteristics and append them to the energy demand record. The data fusion described is typically devoid of uncertainty (as long as the appropriate data processing steps are employed) and well defined as there are attributes that can be used to match data across these multiple datasets. Any data analysis in recent years includes such simple data fusion procedures.

The proposed research is geared towards fusing databases that are not relatable because of the inherent differences across these datasets. For these *uniquely unmatched datasets*, there is a significant need for a behavioral data fusion approach across various domains including energy demand analysis [10–14], mobility pattern analysis [15–17], freight movement modeling [18–20]; disaster evacuation planning [21] and traffic safety [22]. With increasing share of energy use for mobility (with EVs), it is important to examine how transportation mobility needs can influence energy use. The current research recognizes the potential relationship between energy and transportation datasets and provides an algorithm to enhance energy data modeling using information from transportation datasets. The proposed approach is general and can be applied across domains. With emerging advances in information technology and communication devices data from smartphone location data or cell phone OD data are ideal complements to traditional data by offering improved spatiotemporal coverage [23,24]. At the same time, these data are not usually available with person or household level characteristics. Thus, adoption of these data at a decision maker level would require an effective algorithm that can fuse this information with travel survey data.

### 1.2 Research approach

The data fusion algorithm developed in the current research is targeted toward datasets that contain information that is not uniquely matchable. Consider data from a Residential Energy Consumption Survey (RECS) data compiled by US Energy Information Administration (EIA) that provides energy use information by fuel type (such as electricity and natural gas) at a residential unit resolution along with household level information. To understand the determinants of energy use by fuel type, a linear regression model can be estimated using the set of independent variables available in the RECS dataset including household level characteristics: housing type, housing characteristics such as number of stories and bedrooms [25,26]; location characteristics: census region, division, located in urban/rural area [27,28]; and climatic characteristics: number of cooling and heating days [29–31]. However, the RECS data—*source dataset*—does not have any information on the number of employed individuals and household vehicle ownership. It is possible that these two variables are contributing factors for energy use. Employment status and vehicle ownership are indicative of the mobility needs of the household influencing energy consumption at the residence and for transportation needs. The proposed research develops methods that bring in this relevant information from another dataset–a *donor dataset*. The National Household Travel Survey (NHTS) administered by Federal Highway Administration (FHWA) surveys travel behavior patterns. NHTS dataset provides information on employed individuals and vehicle ownership–information that might assist in better understanding energy use and its prediction. With a focus on improving the model for the dependent variable of interest from the RECS dataset (energy use by fuel type in the example), our proposed research provides a probabilistic mechanism for appropriately fusing records from the NHTS dataset with records in the RECS dataset. For each RECS record, the algorithm considers a select set of records from the NHTS dataset with some common attributes (such as census region or household size) as a starting point for matching consideration. A weight function is defined that optimizes the weight for each RECS record while improving dependent variable model fit (energy use by fuel type). As the weight is unobserved to the analyst, the weight function proposed is analogous to the latent segmentation weight for a discrete outcome variable. In our research, the weight function is scored based on the similarity/dissimilarity of the source and donor records for common unmatched attributes (such as number of adults). The weight score is expected to be higher for source and donor records with more similarity. Across the selected donor records for a single source record, the weight sums to one. The donor records selected will provide additional useful variables missing for the source record.

The proposed fusion approach is illustrated using RECS and NHTS datasets for energy use by fuel type analysis. The model developed offers improved data fit for the dependent variables of interest. The main motivation behind our matching approach is to augment RECS data with NHTS data that contains detailed socio- demographics (gender, age), travel patterns (what mode is used for daily travel) and location information that could significantly affect energy usage. For instance, households situated in high population density locations typically have reduced floor area per capita and hence are likely to use less electricity for heating and cooling. Further, in recent years, energy consumption patterns are affected along two directions. First, the emergence of electric vehicles (EV) will transform the energy-transportation relationship. In the future, in households with EVs the energy consumption will be directly associated with vehicle ownership variables (how many electric cars) and vehicle usage dimensions. Second, during the COVID pandemic, a large number of workers facilitated by advances in information technology started to work from home influencing residential energy consumption. Currently RECS data does not provide any information on these important variables. NHTS data on the other hand can fill this gap as information on the number of vehicles in the HH, the corresponding vehicle types (fuel/electric) and the number of people working from home are available. Thus, the proposed fusion algorithm enables us to merge these two distinct datasets and create an enriched data source for analyzing energy consumption. Using the fused data, the association between additional categories of exogenous variables with residential energy demand can be tested. Thus, the model developed with the fused database will have additional explanatory power relative to the model developed solely using RECS data.

The rest of the paper is organized as follows: Section 2 provides a brief review of previous research on the application of data fusion algorithms in transportation field and highlights the contribution of the current study. Section 3 briefly outlines the methodological framework used in the analysis while a detailed description about the experimental setup of the study is presented in section 4. In section 5, we describe the model findings and finally, concluding thoughts are presented in section 6.

## 2 Earlier research and current study

In our research, we are interested in developing advanced approaches for energy consumption analysis drawing on novel approaches from data fusion literature. Hence, we focus our literature review along two directions. In the first direction, we provide a summary of studies examining residential energy usage. In the second direction we provide a summary of studies adopting data fusion techniques in the energy domain.

### 2.1 Literature on energy usage

Residential energy demand has been extensively researched in the energy analysis literature. However to conserve on space, we will provide a brief summary of these studies (see [31] for details on these studies). From our literature review, it is observed that earlier research focused on electricity and natural gas consumption [25,26,29–36] while very limited attention has been devoted to other forms of energies including fuel oil and LPG [31,32,37]. Interestingly, RECS is the most used database in United States for analyzing the usage of various energy sources [29–34]. Within these studies, the most prevalent form of energy usage considered is the continuous representation of energy use including energy consumption in BTU, or natural logarithm of energy consumption [29,30,33,34] while a handful of research efforts focused on the choice of energy source [30–32,34]. Given the continuous nature of the choice variable, it is not surprising earlier research adopted the regression framework for examining the energy usage. In particular, work in this area has ranged from simple linear regression [29,30,33,34] or discrete continuous models [30,34] to more advanced models such as the Multiple Discrete Continuous Extreme Value (MDCEV) model [31,32] for predicting the residential dwelling energy usage. In terms of the predictors, previous studies identified the following factors significantly affecting the residential energy usage: household level characteristics (HH income, race, household size, education) [25,31,36]; location characteristics (census region, type of location) [26,32], housing characteristics (such as year of construction, housing type, type of unit, square footage, and number of stories) [31,35,37], appliance use (such as appliances used in the housing unit) [31,38] and climatic characteristics (such as heating degree days and cooling degree days) [29–33,35].

### 2.2 Literature on data fusion techniques in energy

Data fusion algorithms have been widely researched and applied in various fields including statistics, business analysis, chemical engineering, energy demand, navigation industry and transportation [9,11,19,22,39,40]. For the current research effort, we have confined our attention to the studies adopting data fusion techniques in energy demand sector.

Energy efficiency (in building) is a heavily researched area where data fusion is applied at various resolutions. However, unlike transportation field, data fusion algorithms in energy demand literature mainly focused on appliance, sensor and semantic level fusion as opposed to data level fusion [14]. Example includes system identification combined with Kalman filtering [41], and deep learning-based techniques [11,42] that integrate data from multiple sources. These techniques have been applied to various types of data, including weather, occupancy, and equipment usage patterns. Multi-information fusion models, such as those using convolutional neural network (CNN) and long short-term memory (LSTM) networks, have also been used to enhance the accuracy of energy forecasting [43,44]. Based on the dimension of crucial interest, these studies can be broadly classified into two groups: 1) examine the occupancy status of the building and 2) understand the energy consumption pattern. The reader would note that data fusing algorithms have also been developed to minimize the variance of the fused data, which is beyond the scope of the current study (see [45,46] for details).

The first group of studies mainly adopted different data fusion algorithms for analyzing the occupancy status of a building, a crucial component in energy efficiency and energy consumption analysis [10,11,47–49]. For instance, Wang and his colleagues [47] considered K-Nearest Neighbour (KNN), Support Vector Machine (SVM) and Artificial Neural Network (ANN) algorithms to fuse the environmental data with WI-FI data for predicting the building occupancy. Another research effort by Nesa and Banerjee [48] presented Internet of Things (IoT) based real time sensor data fusion using the data collected from various sensors within office space to predict the occupancy status of the office spaces. Varlamis and his colleagues [10] fused sensor-based energy data with the historical data and user feedback to generate recommendations for smart homes and offices. Wang et al.,[11] used Long Short-Term Memory (LSTM) networks to fuse data from various utilities to predict internal heat gains for office buildings—a major component in heating, ventilation, and air conditioning (HVAC) operations. He et al. [49] proposed the fusion of LSTM and Back Propagation Neural Network (BPNN) algorithms to predict air conditioning load in buildings. Tan and his colleagues [43] employed rule-based decision-making algorithms to combine data from multiple sensors, such as motion, door, and light sensors to improve occupancy detection accuracy in residential buildings.

The second line of inquiry is focused on analyzing the energy consumption patterns of buildings by applying data fusion techniques [12,13,50,51]. Gouveia [13] fused the electricity consumption data from smart meters with door-to-door surveys to understand the energy patterns of the households. Wijayasekara and Manic [51] used ANN based data fusion method to increase the temporal resolution of building energy consumption data. Similar approach was also used by De Silva and his colleagues [50] to understand the energy consumption patterns in buildings. Gurino et al.,[12] compared the existing climatic databases with the simulated historical weather data aimed to generate a fused dataset by using various climate change models. This fused database was used to predict the consumption of energy requirements for office buildings.

### 2.3 Current study in context

The literature review clearly highlights the prevalence of data fusion algorithm across various energy sectors. However, all these studies focused on combining two/more datasets based on a common identifier (such as fusing information to a house based on its ID) or by employing black box approaches to data fusion. Furthermore, the data fusion approaches are geared towards compiling dependent variables of interest not available in one of the datasets. In our research, the focus is on providing additional independent variables for accurately representing the dependent variable of interest. The preceding discussion also makes it clear that data fusion algorithms in energy demand literature are primarily focused on semantic, sensor, and appliance level fusion, as opposed to observation level probabilistic fusion approach proposed in our study [14]. To the best of the authors’ knowledge, this is the first attempt (in both transportation and energy demand literature) to develop a behavioral fusion algorithm to combine two different datasets without any common identifier. A recent paper by Zhang and his colleagues (60) adopted a fusion approach to predict credit risks for small and medium-sized businesses (SMEs) in supply chain financing by merging behavioral and demographic data. However, the work also focused on deterministic fusion as both these data were matched based on the common entity of SMEs in supply chain finance.

The current approach is focused on a data fusion approach that augments RECS data (source) with additional variables from NHTS dataset (donor) with a focus on improving the data fit of the dependent variable of interest (energy use by fuel type) in the source dataset. The source and donor dataset can have common attributes such as census region, household size, household ownership, number of adults, and area (urban/rural). Ideally, selecting all or the majority of the common attributes for matching would provide the most precise fusion. However, the reader would recognize that selecting all or a large number of common attributes as matching variables can potentially reduce viable matching candidates or result in zero candidates. This would have resulted in the loss of records and potentially introduced bias, as significant portions of the dataset might be excluded from the analysis. Hence, we employ an approach where we choose a subset of common attributes for matching. As the matching between source and donor sets are being considered across different datasets, we hypothesize that fusing multiple candidates (as opposed to one record) would allow for a more useful and representative fused dataset. At the same time, as we fuse multiple records (say K) from the donor dataset (NHTS) with the source dataset (RECS), the source record will need to be duplicated K times to generate fused records. To address this duplication, a simple *deterministic* weight (1/K) is applied to ensure for each source record, the multiple matched rows of data represent only one new record. The proposed fusion approach makes several variables that are not available in the original dataset accessible for modeling. The benefit from these additional variables can be evaluated in a straightforward manner. If these additional variables contribute to improving the data fit of the dependent variable, then the fused dataset offers improved analysis of the dependent variable of interest. The improvement in data fit is compared using the log-likelihood and Bayesian Inference Criteria metrics that are well established in the literature.

The deterministic matching approach will work effectively with a small set of matching variables. As the number of potential matching variables increases, the number of exact matches could reduce very quickly. Therefore, we propose a matching approach with a *probabilistic* weight that penalizes differences between the source record and the donor record. So, in this approach, we allow for some variable mismatch and evaluate its impact on matching process by estimating a weight for each donor record that is fused with a source record. Specifically, the weight is parameterized as a function of the discrepancy for variables in both datasets. The contribution is influenced by similarity (or dissimilarity) across the common attributes between source and donor datasets. This weighting process effectively translates to estimating the weight contribution of the donor record to improve data fit of the dependent variable of interest (as opposed to using a uniform 1/K weight). The records with smaller mismatch are likely to have a weight higher than the deterministic weight (1/K) and records with higher mismatch are likely to have a weight lower than the deterministic weight. The parameters estimated as part of the weight function will inform us about the ranking of the various matching factors on their impact on the dependent variable of interest. For instance, household ownership status might not be as important as number of children in explaining household energy consumption patterns. In this case, the weight function coefficient for difference in the number of children variable will be larger in magnitude.

**Fig 1:**
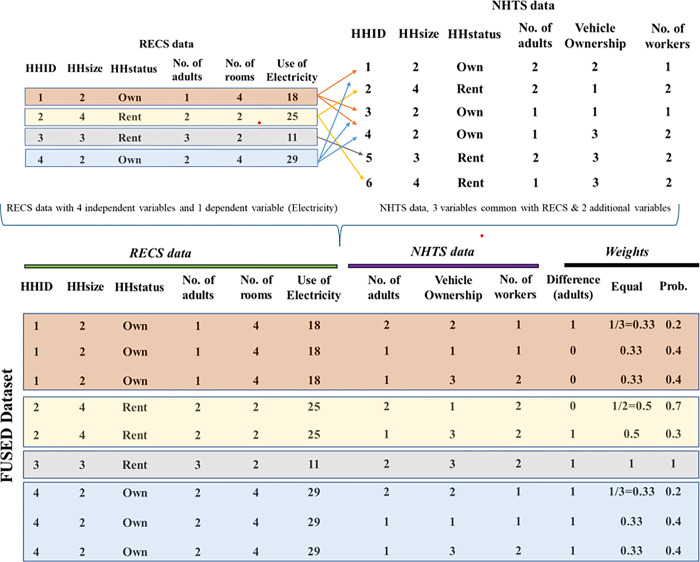
RECS and NHTS data fusion illustration.

To better illustrate the data fusion process, an example is presented in Fig 1. The RECS Survey has four HHs with information on household size, household ownership status, number of adults in the HHs, number of rooms in the HH and the dependent variable: consumption of electricity (in millions of Btu). The NHTS data, in addition to household size, ownership status and number of adults, provides information on vehicle ownership and number of workers in the HH. The common variables across these two datasets are household size, ownership status, and the number of adults. Initially, we begin the fusion using all three matching attributes. In this process, we are able to find matches for all households except the third household. If we proceed with this fusion, then the third household would need to be excluded from the analysis, thereby compromising 25% of the records (1 household out of 4 households in RECS). To address this issue, we relax the matching assumption by considering two variables (household size, and household ownership status) as our matching attributes while use the remaining variable (number of adults) in the weight function. Based on this, we find three matches for the first HH, two matches for the second household, one match for the third household, and three matches for the fourth household. Now, using the matched records, a fused dataset is created with three repetitions of HH 1, two repetitions of HH2, 1 HH3 and three repetitions of HH4 with NHTS data columns including number of adults, vehicle ownership and number of workers in the HH (see Fig 1). As mentioned earlier, a weight function is used in the data to ensure that all the repetitions together represent one household in the RECS data. For the deterministic weight method, we assign an equal weight, that is 1/K for K repetitions. For example, for HH 1, which has three repetitions, each repetition would be assigned a weight of 1/3 (approximately 0.33). For the probabilistic weight method, we will calculate the difference in the number of adults variable (available in source and donor datasets but not matched) across the two datasets and use these differences to parameterize the weight function (details on this process is discussed in the methodology section). The probabilistic weight variable provides a higher weight when the difference is lower (or 0. For example, for HH 2 (see Fig 1), the first matched record has the same number of adults as the RECS dataset, resulting in a higher weight of 0.7. In contrast, the second matched record does not have the same number of adults, resulting in a lower weight of 0.3. Please note that the numbers provided in Fig 1 are for illustration purposes and will be estimated in our model within a maximum likelihood setting.

In summary, the current study contributes to the energy and data science literature both empirically and methodologically. Empirically, the proposed fusion algorithm enables us to merge these two distinct datasets and create an enriched data source for analyzing energy consumption. Using the fused data, the association between additional categories of exogenous variables with residential energy demand can be tested. Thus, the model developed with the fused database will have enhanced explanatory and predictive power relative to the model developed solely using RECS data. Further, this enriched dataset, and the resulting model can significantly inform policy decisions. For example, understanding the impact of EV ownership and working-from-home trends on residential energy consumption can guide policymakers in designing targeted incentives for energy-efficient technologies and infrastructure. Methodologically, the study presents an innovative behavioral data fusion technique to combine two datasets without a common identifier. Further, our approach strategically selects variables for initial matching and incorporates the remaining ones into a weight function, ensuring an optimal balance between sample size and important variables. This type of behavioral fusion is introduced for the first time in this paper (to the best of the authors’ knowledge) and can be widely applied to various fields.

## 3 Experimental design

The objective of the current research effort is to illustrate how we can fuse two disparate datasets to enhance the model development for a dependent variable present in the RECS dataset using variables from the NHTS dataset. In the presence of a set of common variables, the fusion process will be affected by several aspects: (1) how many deterministic matching variables will be used and how many probabilistic matching variables will be used, (2) how many records from the donor dataset will be fused with each source record and (3) how will we assess the impact of randomness of fusion process on parameter stability.

**Fig 2:**
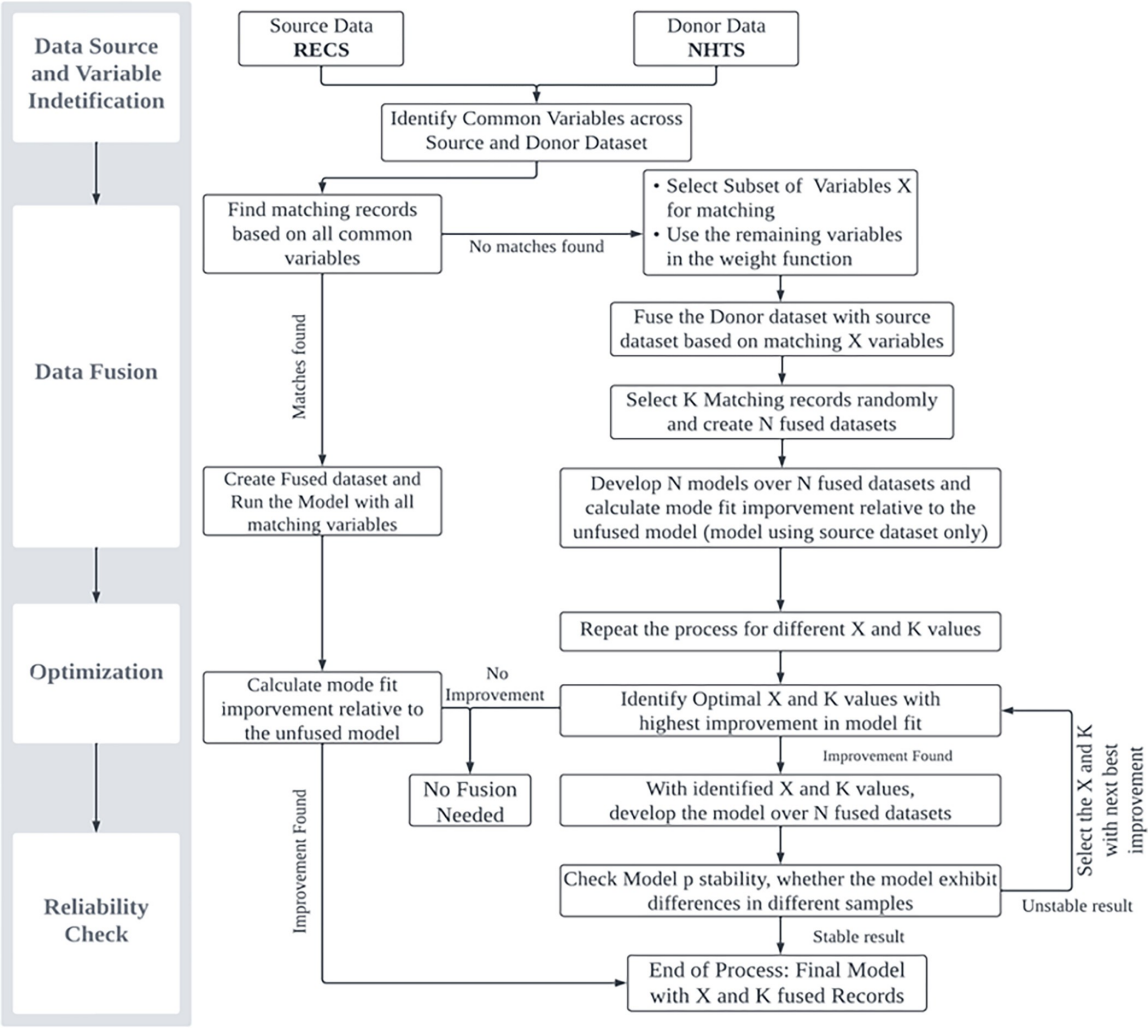
Flow chart showing research framework for the fusion algorithm.

In this section, we present an experimental setup documenting the structure of how the fusion process will be tested (see Fig 2). The overall process consists of four stages: Data Source and Variable Identification, Data Fusion, Optimization, and Reliability Check. The initial stage involves identifying the two datasets for fusion: the source dataset that serves as the primary dataset for analysis and the donor dataset from which additional information will be incorporated. After identifying the two datasets, we will determine the common variables between them: these are the variables that form the basis of matching the source and donor dataset. The next stage is the Data Fusion, where the process begins by checking if it is possible to fuse the two datasets based on all common matching variables. If substantial matching records can be found for each record in the source dataset, the datasets are fused, and the model is developed. However, if matching records are insufficient, then a subset of those common variables is selected for the matching process, and the remaining variables are used in the weight function to allow for probabilistic matching. When selecting the subset of variables, different combinations can be used for fusion, including a single variable (e.g., matching by HH size) or variable groups comprising multiple variables (e.g., matching by HH size and location). Based on the matching variables, we can identify potential candidates from donor dataset that can be appended to each source record. The matching process can result in a several records for each source record (say M). Hence, selecting a single record or selecting a set of records randomly might introduce bias. We select a fixed number of records (say K) and repeat the sampling process several times (say N = 15). For example, let’s say we match RECS and NHTS based on HHsize and number of adults. By doing so, we find 100 potential matches (M = 100) for each RECS HH from the NHTS dataset. However, estimating models with all these fused records increases the model estimation burden. Hence, we start our fusion process by fixing the number of matching records to be 5 (K = 5) and generate 15 mutually exclusive samples (N = 15). Now, with these samples established, we run N number of models for all the samples with new variables from the NHTS dataset (donor dataset) and evaluate if the average model fit in terms of log-likelihood has improved relative to the model estimated on the RECS dataset only (source dataset).

The process then proceeds to the Optimization stage. During this stage, the data fusion process is repeated multiple times with varying matching variable combinations to determine which variables offer the best improvement over the non-fused model. The variable (or variable combination) that offers the most significant improvement is identified as the optimal matching variable (or variable combination). The next step is to determine the optimal number of records to be matched between the source and donor datasets. For the selected X matching variable (or matching combination), the process tests if changing K (3, 5, 10, 15, 20, 40, 50) affects the average log-likelihood improvement. The examination ensuring that the improvement is not a random occurrence and is consistent for different numbers of matched records. The K providing the highest improvement is selected, representing the optimal number of matched records for the fusion process. Once the optimal X and K values are identified, the next step is to check the robustness of the fusion process, which is conducted in the final stage of the experimental setup named Reliability Check (see Fig 2). It is possible that the model developed from the fused dataset based on X and K can differ from the model developed on a different sample with the same X and K, due to the random selection of K records. For instance, if 10 records are identified as the optimal match out of 100 possible matches, the first sample might include a randomly selected set of 10 records, while a different set of 10 records might be selected for the second sample. Consequently, the models developed from these two samples could vary significantly. If substantial differences are observed between the models, it indicates that the results are highly dependent on the randomness of the selection process. Therefore, ensuring the reliability of the fusion process is crucial to validate the stability and robustness of the model outcomes. To check this, we generate S number of samples (S = 25) considering the selected X and K and develop the same model for all S samples. After that, we evaluate the consistency of the models at a parameter level i.e., we check if the parameters remain stable across all S samples of the data for that K. To be specific, we compare the models across the S samples using an approximate t-test to see if these parameters vary across the samples. If we find any variation across the samples, then it lends evidence to instability in parameter magnitudes and signs. Therefore, that corresponding X is excluded from the fusion process, and we proceed to test the next best combination of X and K. This process is repeated until all criteria are satisfied, ensuring that the identified X and K values lead to consistent and reliable improvements, as confirmed through the reliability check.

## 4 Data description

**Fig 3:**
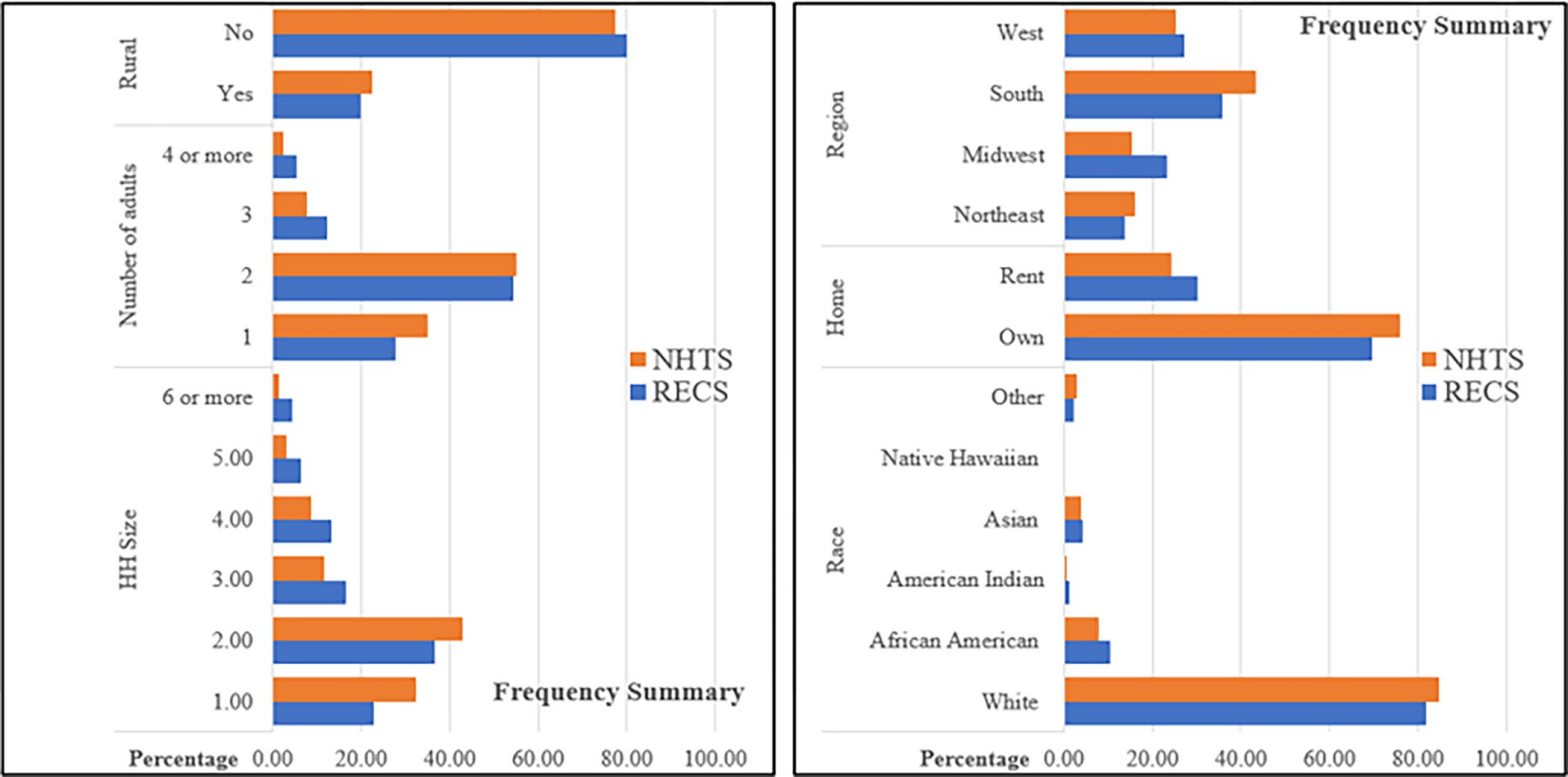
Comparison of household distributions across NHTS and RECS datasets based on common variables.

The dependent variable of interest in our research is energy usage by fuel type (electricity and natural gas) in residential dwellings. The energy use data is drawn from the 2015 Residential Energy Consumption Survey (RECS) administered by US EIA. The RECS data, for 5,686 households, provides detailed information on energy usage, housing characteristics (such as construction period, number of rooms, bedrooms), appliances used (such as internet, mobile phone, number of refrigerators, desktop, use of ac and heater); location related variables (such as census division, area of the household: rural/urban); and climatic variables (such as number of cooling and heating degree days). Out of these 5,686 households, we randomly selected 4,000 households as our estimation sample and the remaining 1,686 households were set aside for validation exercise. Several relevant variables are missing in RECS data such as the number of employed individuals, number of female household members, number of drivers and workers in the household, household vehicle ownership, population density, and daily travel pattern (like use of car, bike, transit, walk on a daily basis). To evaluate the potential value of this information, we employ the NHTS survey data that provides information on the missing variables as a potential donor dataset. The RECS and NHTS datasets share seven variables along two dimensions: HH related factors (such as household size, no. of adults in HH, race and home ownership status) and location related variables (HH region, HH division, HH location classified as rural/urban). Table 1 presents detailed summary statistics for both dependent and independent variables from both RECS and NHTS dataset respectively. Further, before proceeding with the fusion, we checked the distribution of households across the two datasets based on all common variables. The comparison is presented in Fig 3, and as can be seen, the distributions of the households from both datasets are quite comparable, thereby validating the alignment of the datasets for meaningful fusion. This step is crucial as it ensures that the two datasets represent similar populations, minimizing potential discrepancies.

**Table 1:** Dependent and independent variables summary from RECS and NHTS data.

Variable	*Minimum*	*Maximum*	*Average*
**Dependent Variables form RECS**			
Electricity usage (in 10^6 BTU)	0.200	215.69	37.73
Natural gas usage (in 10^6 BTU)	0.000	306.59	33.54
**Independent Variables from RECS**			
*HH Characteristics*			
Total square footage	221.000	8501.00	2081.44
Number of bedrooms	0.00	10.00	2.83
Total number of rooms	1.000	19.00	6.19
Housing type—Mobile home	0.00	1.00	0.05
Housing type—Apartment	0.00	1.00	0.66
Construction year 1981–2000	0.00	1.00	0.29
Construction year 2001–2010	0.00	1.00	0.16
Construction year after 2010	0.00	1.00	0.04
High income HH (>120k)	0.00	1.00	0.15
*Appliance Use*			
AC Used	--	--	0.87
Number of refrigerators used	0.00	8.00	1.40
Number of desktop computers	0.00	10.00	0.52
Space heating used	0.00	1.00	0.95
Number of smart phones	0.00	8.00	1.60
Humidifier used	0.00	1.00	0.20
*Climatic Variables*			
Total cooling degree days, base temperature 65F	0.00	6607.00	1719.21
Total heating degree days, base temperature 65F	0.00	9843.00	3707.85
**Independent Variables from NHTS**			
Population Density			
Medium	0.00	1.00	0.21
High	0.00	1.00	0.06
Number of females in HH	0.00	8.00	1.09
Number of vehicles in HH	1.00	12.00	2.11
Number of drivers in HH	0.00	9.00	1.77
Number of workers in HH	0.00	7.00	1.08
Mean age of HH members	11.00	92.00	52.87
HH average annual miles	2.83	254,309	20,994
People use car daily	0.00	1.00	0.16
People use bicycle daily	0.00	1.00	0.01
People walk daily	0.00	1.00	0.16
People use transit daily	0.00	1.00	0.01

### 4.1 Selecting variables fusion

**Fig 4:**
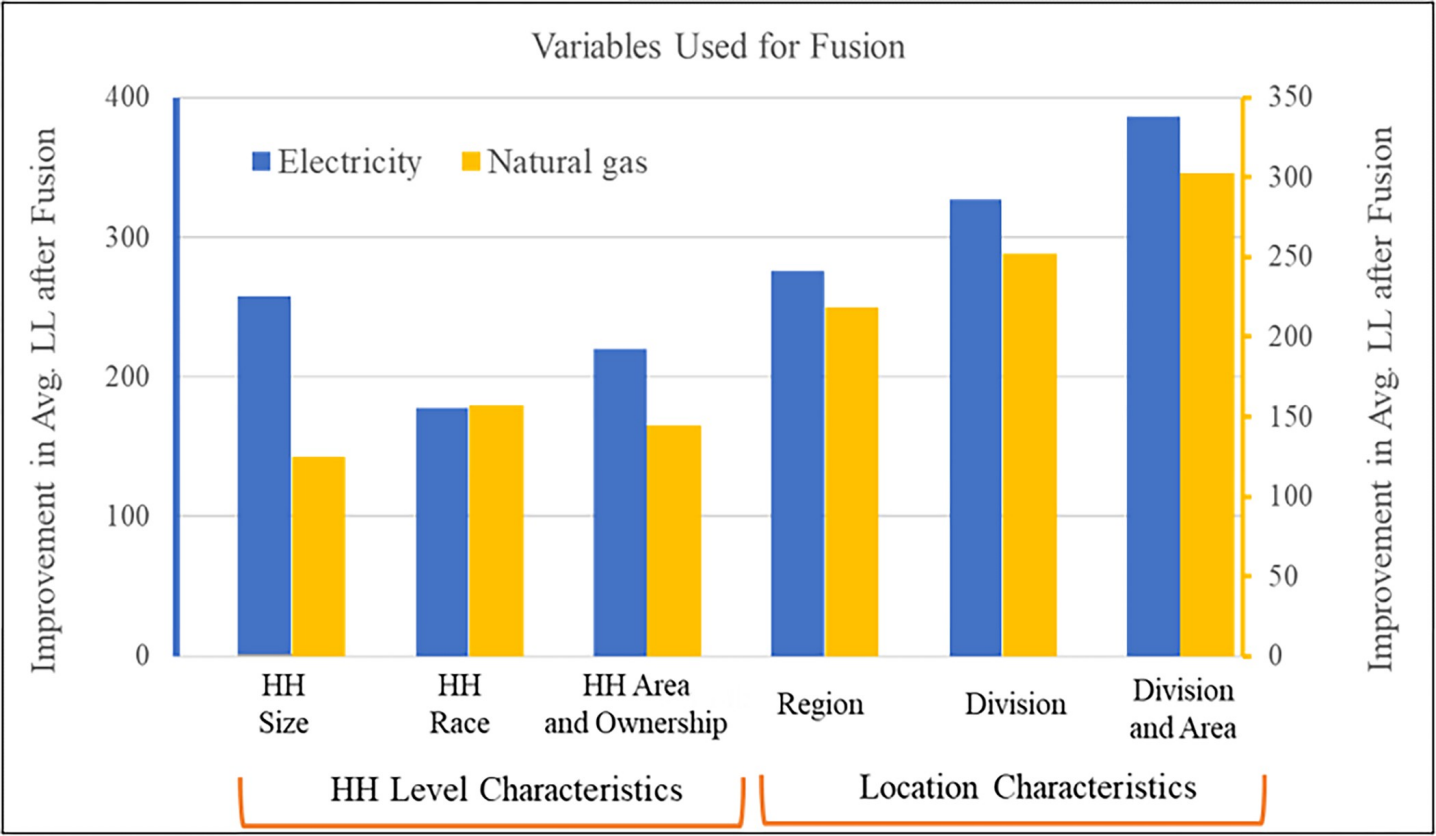
Model fit summary across different variable group used for fusion.

In the current analysis, we tried several combinations of these factors for linking the two datasets and for each combination, we calculate the improvement in average (we consider N = 15 samples) log-likelihood (LL) relative to the simple linear regression model that is estimated using the RECS data only. Finally, we select the corresponding combination that provides the superior improvement. The average LL improvement measures across each variable/variable groups are plotted in Fig 4. From this plot, we can clearly see the relatively higher average LL improvement when household from both datasets are fused based on census division and location classified as urban or rural. We select this variable group for linking the two datasets and proceed to the next step.

### 4.2 Selecting number of matching records for fusion

**Fig 5:**
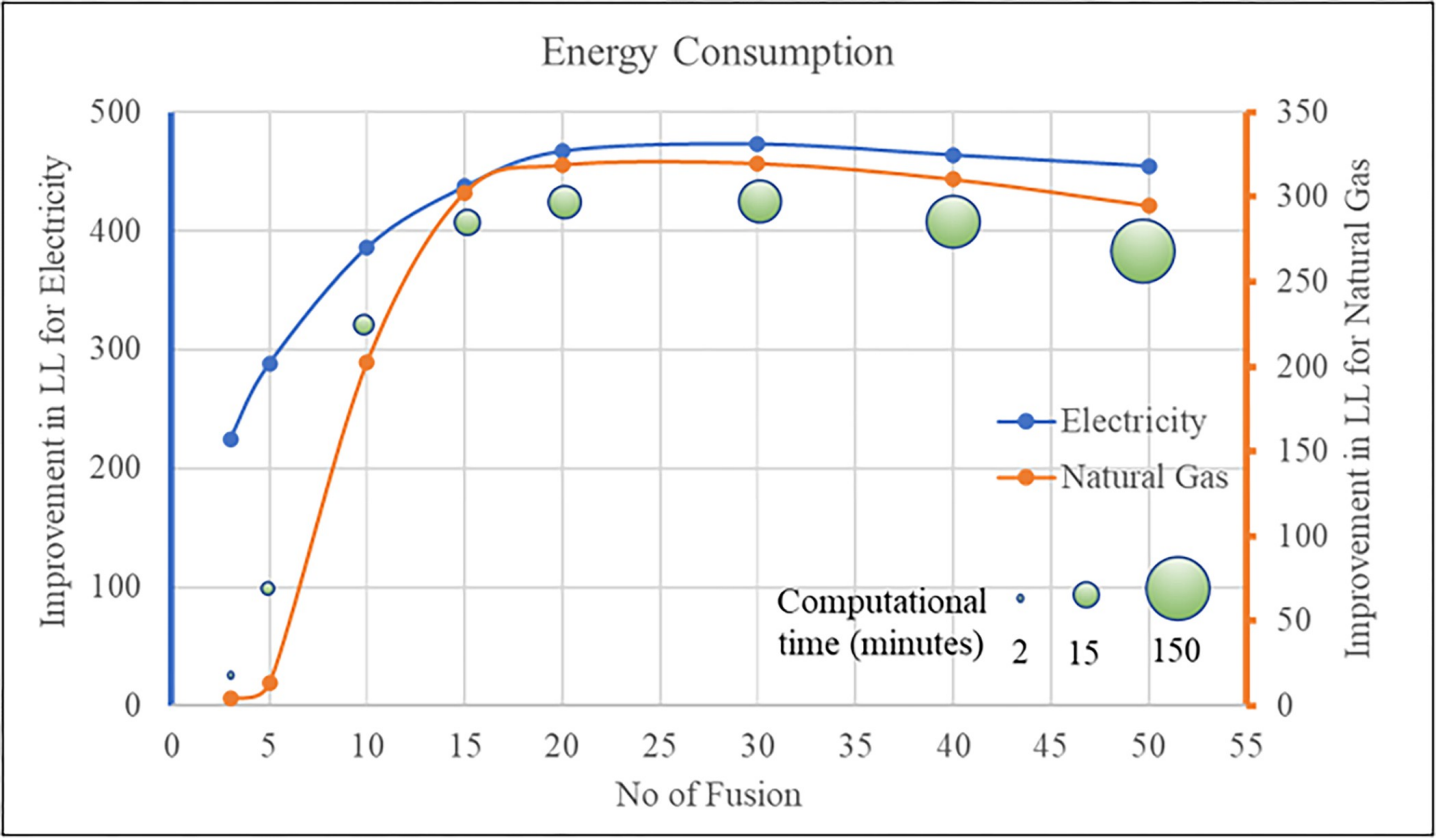
Model fit summary across different number of fusion.

Based on the result obtained in the first step, we linked the two datasets based on similar HH location and created N = 15 fused databases using multiple matching records of K including 3,5, 10, 15, 20, 30, 40 and 50 (see Fig 5). We compute the improvement in average LL measures for different values of K. From the Fig 5, we can clearly see there is significant improvement in average LL as K increases in the initial stages. After a K value of 15, only marginal changes to average LL improvement are noticed. However, with increased K value, the model estimation times will continue to increase as the number of effective records increase with K. Thus, from the perspective of model improvement and run times, we select K = 15 as the optimal value. Thus, for each sample, 15 records from NHTS will be added to the RECS sample.

### 4.3 Check parameter estimates stability

After selecting the variables and the number of records to be used for fusion, the next step is to evaluate the stability of the parameters of the energy demand model estimated using the fused data. As described, multiple samples were generated for the fused dataset, and it is important to confirm that the parameter estimates from all these samples offer consistent results. To undertake this evaluation, we propose an approximate t-statistic measure for each sample parameter estimate as follows:

$$t_{s}=Abs(\frac{{(B}_{m}-B_{s})}{\sqrt{SD_{m}^{2}+SD_{s}^{2}}})$$ (1)

**Fig 6:**
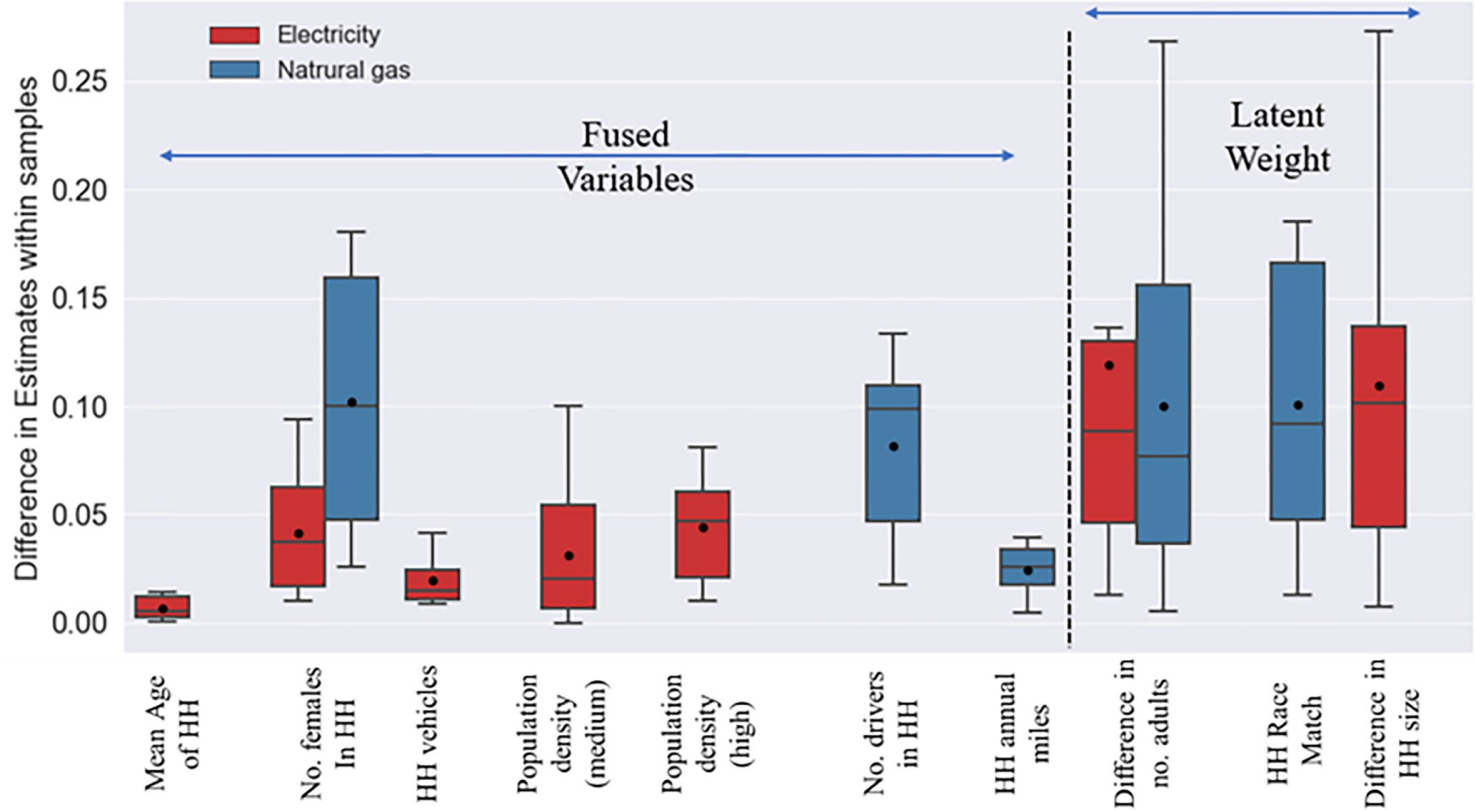
Test statistics (t-statistics) for parameter estimates across samples for each variables and models.

Where *B*_*m*_ is the average estimate value across all N samples ($B_{m}=\frac{1}{N}*\sum_{s=1}^{N}B_{s}$); *B*_*s*_ is the estimate for the *s*^*th*^ sample; *SD*_*m*_ is the average standard error for all N samples ($SD_{m}=\frac{1}{N}*\sum_{s=1}^{N}{SD}_{s}$) and *SD*_*s*_ is the standard error for the sth sample. If the computed t-statistic value is greater than 1.65 it indicates that the parameter estimate is quite different from the average parameter across the samples. The t-statistic across all parameters and samples can be computed and used to measure the number of outliers. The presence of outliers will indicate that significant parameter variability across the samples and hence the results are less likely to be stable in this case. In our study context, we computed the approximate t-statistic for fused model parameters in the energy use component and parameters in the weight component. The results are plotted in Fig 6. The boxplots clearly illustrate significant stability in the parameters estimated. In fact, the computed approximate t-statistic does not reach 1.65 for even one parameter across all samples. The highest single value obtained is under 0.3, while the mean values range around 0.1. The results clearly indicate that for the fused dataset, we have obtained a reasonably stable estimate for all parameters.

## 5 Methodology

In this section, we will present the methodological framework adopted in the study for analyzing the residential energy usage.

The model structure estimated in the current research effort has a choice model component (energy usage) and a weight component. In the choice model component, we consider the natural logarithm of the energy usage (separately for electricity and natural gas) as our dependent variable and employ linear regression model for analyzing the continuous outcome variable.

Let us assume that there are *i* (1.2,…N, N = 4,000) HHs in RECS survey data and *K* possible matches from the NHTS dataset. *d* be an index to represent the residential energy usage by different sources (electricity and natural gas). Let *y*_*d*,*i*_ and *Q*_*d*,*ik*_ is the observed and predicted lognormal of the energy usage in HH *i* for the *K*^*th*^ fused records by energy source *d* respectively (the *y*_*d*,*i*_ will be same across all the *K* fused records for HH *i*). In the current study context, separate linear regression models are estimated for electricity and natural gas consumption and hence *d* is omitted in the following equations for simplicity. Following this, the formulation of the linear regression model can be written as:

$$Q_{ik}=\beta^{\prime }X_{ik}+{\gamma^{\prime }S}_{ik}+\varepsilon_{ik}$$ (2)

where, *X*_*ik*_ is a vector of attributes from the source dataset that influence energy demand and *β*′ is the corresponding coefficients to be estimated (including a scalar constant). *S*_*ik*_ is the vector of attributes from the donor dataset that affect energy demand and *γ*′ is the corresponding vector of coefficients to be estimated. The reader would note that to estimate the unfused model using source data only, we restrict *S*_*ik*_ to be empty. *ε*_*ik*_ is independently and identically distributed error term with zero mean and variance *σ*^2^. Based on this, the probability for HH *i* for the *K*^*th*^ fused records to have *y*_*i*_ energy demand is given by:

$$P(Q_{ik})|\beta^{\prime }{,\gamma }^{\prime }=\frac{\phi [\frac{y_{i}-Q_{ik}}{\sigma }]}{\sigma }$$ (3)

where ϕ(.) is the standard normal probability density function.

On the other hand, the weight component takes the form of a latent multinomial logit structure (MNL) allocating the probability for each RECS HH being paired with an NHTS HH. The matched weightage propensity is determined based on a latent probability value estimated using a multinomial logit model as follows:

$$P_{ik}=\frac{\exp\left(\propto Z_{ik}\right)}{\sum_{k=1}^{K}\exp\left(\propto Z_{ik}\right)}$$ (4)

where *Z*_*ik*_ is a vector of attributes considered for matching, ∝ is a corresponding vector to be estimated. Based on this notation, let’s assume *Q*_*i*_ is the weighted probability that HH *i* has *y*_*i*_ energy demand which can be written as:

$$Q_{i}=\sum_{k=1}^{K}P(Q_{ik})xP_{ik}$$ (5)

This matching, when executed, will provide us a relationship between the RECS and NHTS datasets. Specifically, employing Eq 5, several additional variables from the NHTS dataset will be employed to generate the missing dimension for the RECS dataset. Finally, the log-likelihood function for the fused dataset energy demand is defined as:

$$LL=\sum_{i=1}^{N}log(Q_{i})$$ (6)

## 6 Empirical analysis

### 6.1 Model fit

The experimental set up and the corresponding results establish the best model estimated using the fused dataset. We estimate multiple models to serve as a benchmark for the proposed models. First, we estimate a simple linear regression model (SLR) employing the RECS survey (with 4,000 HHs) data without fusing any record from the NHTS database. Second, we employ the fused dataset with K = 15 and N = 15 and estimate a linear regression model with equal weights (EWLR) allocation i.e. each fused record is weighted at (1/15). Finally, these two models are compared with the fused latent weight linear regression (LWLR) model. The models are estimated for two use cases: electricity energy use and natural gas energy use.

The performance of these models is compared based on the log-likelihood (LL) at convergence, the number of parameters estimated, and Bayesian Information Criterion (BIC). For the electricity demand model, the BIC (LL) values at convergence are: 1) SLR model (with 16 parameters)– 6,126.73 (-2997.01); 2) EWLR model (with 21 parameters)– 5,859.04 (-2814.00); and 3) LWLR model (with 23 parameters)– 5,806.38 (-2776.67). For the natural gas demand model, the values are: 1) SLR model (with 9 parameters)– 9,882.92 (-4891.95); 2) EWLR model (with 12 parameters)– 9,685.34 (-4,776.60); and 3) LWLR model (with 14 parameters)– 9,635.35 (-4740.66). Two important observations can be made from the model fit measures. First, models incorporating additional variable information from the NHTS dataset always provide improved performance irrespective of the dependent variable (electricity and natural gas usage). Second, within the models using fused dataset, the LWLR model outperforms the EWLR model as indicated by the lower BIC value associated with the LWLR model. This result clearly supports our proposed approach that a donor record’s contribution can be optimized using the weight function based on the similarity/dissimilarity of the common attributes. Overall, the model fit measures provide strong evidence for model improvement via fusion as well as weighted contribution estimation.

### 6.2 Estimation results

This section offers a discussion of the exogenous variable effects on energy usage for electricity and natural gas. Results obtained from the final model are presented in Table 2. It should be noted that the final specification of the model development was based on removing the statistically insignificant (90% significance level) variables from the model. A positive (negative) sign in the Table 2 indicates the increased (decreased) energy usage for the corresponding source (electricity/natural gas). The results are presented by variable groups.

**Table 2:** Latent Weight Linear Regression (LWLR) model estimation results.

Variable	Electricity Consumption		Natural Gas Consumption	
	*Estimates*	*t-statistics*	*Estimates*	*t-statistics*
**RECS Data**				
Constant	0.642	3.564	-5.109	-22.914
*HH Characteristics*				
Ln (Total square footage)	0.336	7.269	0.638	9.309
Number of bedrooms	0.060	4.794	0.081	5.133
Total number of rooms	0.028	4.481	--	--
Housing type—Mobile home	0.217	6.065	--	--
Housing type—Apartment	--	--	-0.372	-8.582
Construction year 1981–2000	0.040	1.793	--	--
Construction year 2001–2010	0.049	2.232	-0.097	-2.684
Construction year after 2010	0.012	2.297	-0.392	-5.652
High income HH (>120k)	--	--	0.177	5.149
*Appliance Use*				
AC Used	0.249	10.043	--	--
Number of refrigerators used	0.137	10.776	--	--
Number of desktop computers	0.049	4.228	--	--
Space heating used	0.158	4.148	--	--
Number of smart phones	0.029	4.116	--	--
Humidifier used	-0.107	-5.364	--	--
*Climatic Variables*				
Ln (Total cooled square footage)	0.329	12.997	--	--
Ln (Total heating square footage)	--	--	0.873	20.934
**Variables form NHTS**				
Population Density				
Medium	-0.385	-12.197	--	--
High	-0.631	-16.792	--	--
Number of females in HH	0.069	2.588	0.079	2.842
Number of vehicles in HH	0.041	2.795	--	--
Number of drivers in HH	--	--	-0.047	-1.807
Mean age of HH members	-0.005	-5.176	--	--
HH average annual miles	--	--	0.401	92.361
scale	0.430	51.838	0.553	61.640
**Weight Component**				
HH member difference	-0.636	-5.196	--	--
No. of adult differences	-0.543	-2.785	-0.180	-2.137
HH race match	--	--	0.397	3.164

#### 6.2.1 RECS variables

**Fig 7:**
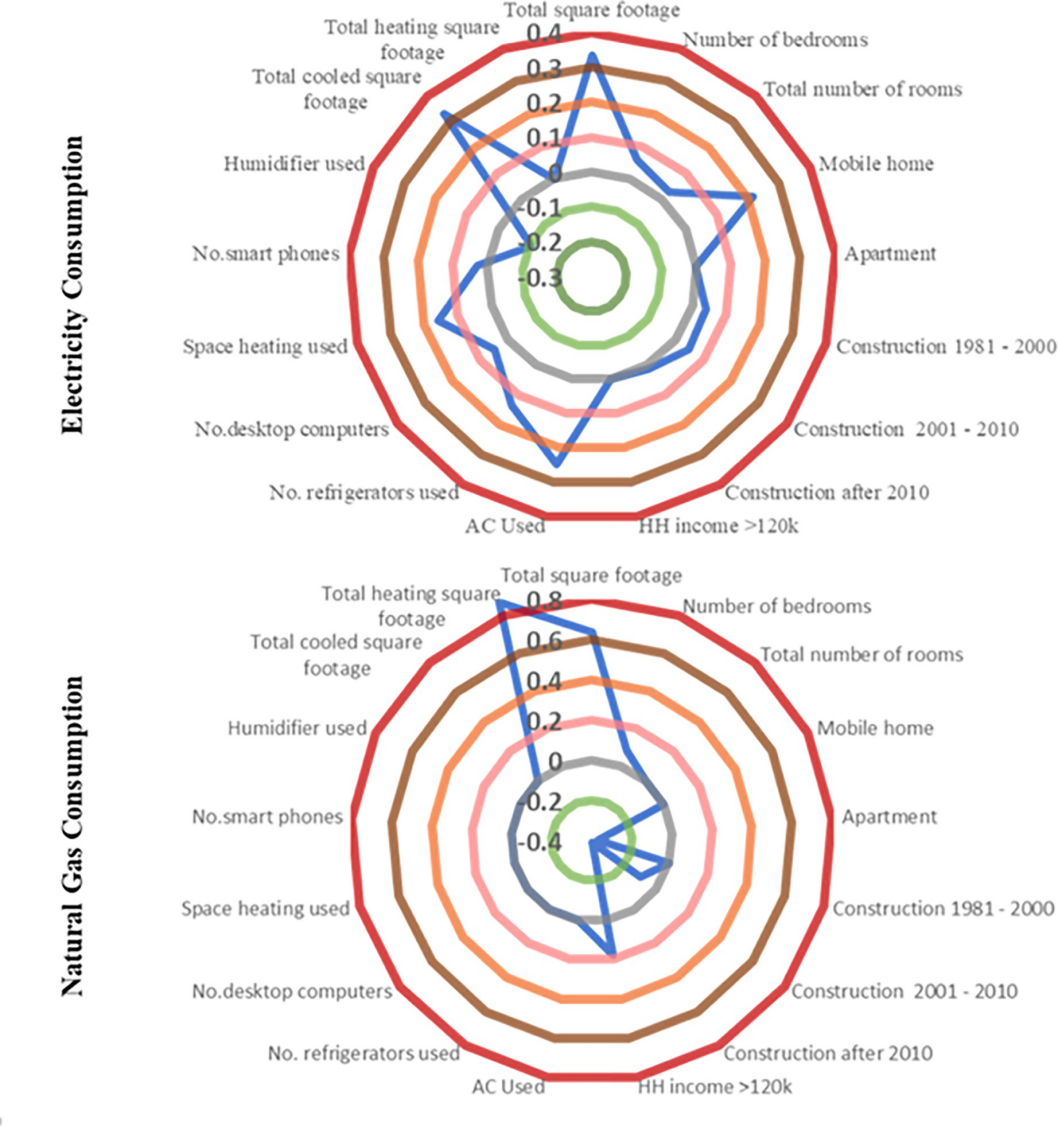
Graphical representation of RECS variables’ impact on energy consumption.

From our analysis, we find significant impacts of several RECS variables on energy consumption, as indicated in Table 1. To better illustrate these impacts for the readers, we present our findings graphically in Fig 7.

Constant: The constant parameter does not have any interpretation after incorporating other variables.

HH Characteristics: In terms of household characteristics, several attributes influence the usage of electricity and natural gas in residential dwellings. For instance, housing unit size (total square footage) reveals a positive impact on energy mix indicating a higher usage of electricity and natural gas in larger houses. This is intuitive as capital costs for installation for non-electricity sources might be high for smaller houses. On the other hand, in bigger houses, a mix of energy sources might be economical in the long run (see [30,32] for similar results). Further, higher number of bedrooms contribute to increased energy usage (both electricity and natural gas) as indicated by the positive coefficient in Table 2. In addition to the bedrooms, we also explored the impact of total number of rooms in a household on energy demand. Interestingly, we find that the variable has a significant positive impact on electricity consumption only. The reader would note that though all these variable seem to be influenced by each other, we did not find any significant correlation across them and thus are simultaneously considered in the model.

The results associated with housing type show significant impact on energy usage. Electricity consumption is likely to be higher in mobile homes while a lower usage of natural gas usage is observed in apartments. The results perhaps indicate inefficient cooling and heating in mobile homes resulting in increased electricity usage [52]. Further, building construction period is also found to have a significant impact on energy consumption. Specifically, we find an increased electricity usage in houses constructed after 1980 relative to the older houses (before 1980) while the natural gas usage is gradually declining in newer houses (after year 2000) as indicated by the negative sign in Table 2. The result is consistent with the overall trend of natural gas consumption in US. Newer buildings are associated with improved insulation, building materials and efficient heating systems contributing to lower benefits from employing natural gas consumption compared to the benefits of natural gas in to older buildings [32,53]. The growing adoption of all-electric homes in recent years is another important factor affecting natural gas consumption [54]. Finally, the income variable highlights a higher natural gas consumption in high-income households (greater than 120k).

Appliance Use: The intensity of appliance use in residential buildings potentially contributes to the overall energy usage. As expected, all of the appliance related attributes (use of ac and space heating; number of refrigerators, computers and smart phones in HH) positively impacted the electricity usage in a house [31] except the variable that corresponds to the use of humidifier. This result (humidifier) while counterintuitive at first glance, is presumably capturing the indirect relationship with the cooling and heating behaviour in a household. For instance, humidifier helps in creating a soothing environment by adding moisture in the air appropriately both in summer and winter season, thus minimizing the need of raising/lowering the temperature in a household [52] and hence possibly reducing electricity consumption.

Climatic Variables: The results related to climatic variables highlight the important role of weather in household energy usage. For representing the climatic variables, we considered heating and cooling degree days (please see [31] for detail) in a household that quantifies the demand for energy needed for heating and cooling requirements of a building respectively. Higher heating and cooling degree days directly refer to the cold and hot weather respectively. As expected, we find electricity usage to be positively associated with cooling degree days revealing an increased electricity consumption during hot days, perhaps alluding to the higher usage of AC during those times [55]. Contrastingly, natural gas consumption is higher during cold weather as evidenced by the positive sign specific to the heating degree days variable. Households in colder regions usually have higher space heating needs and natural gas is one of the predominant sources of fuel for space heaters. Similar findings are also observed in earlier research [31,32].

#### 6.2.2 NHTS variables

**Fig 8:**
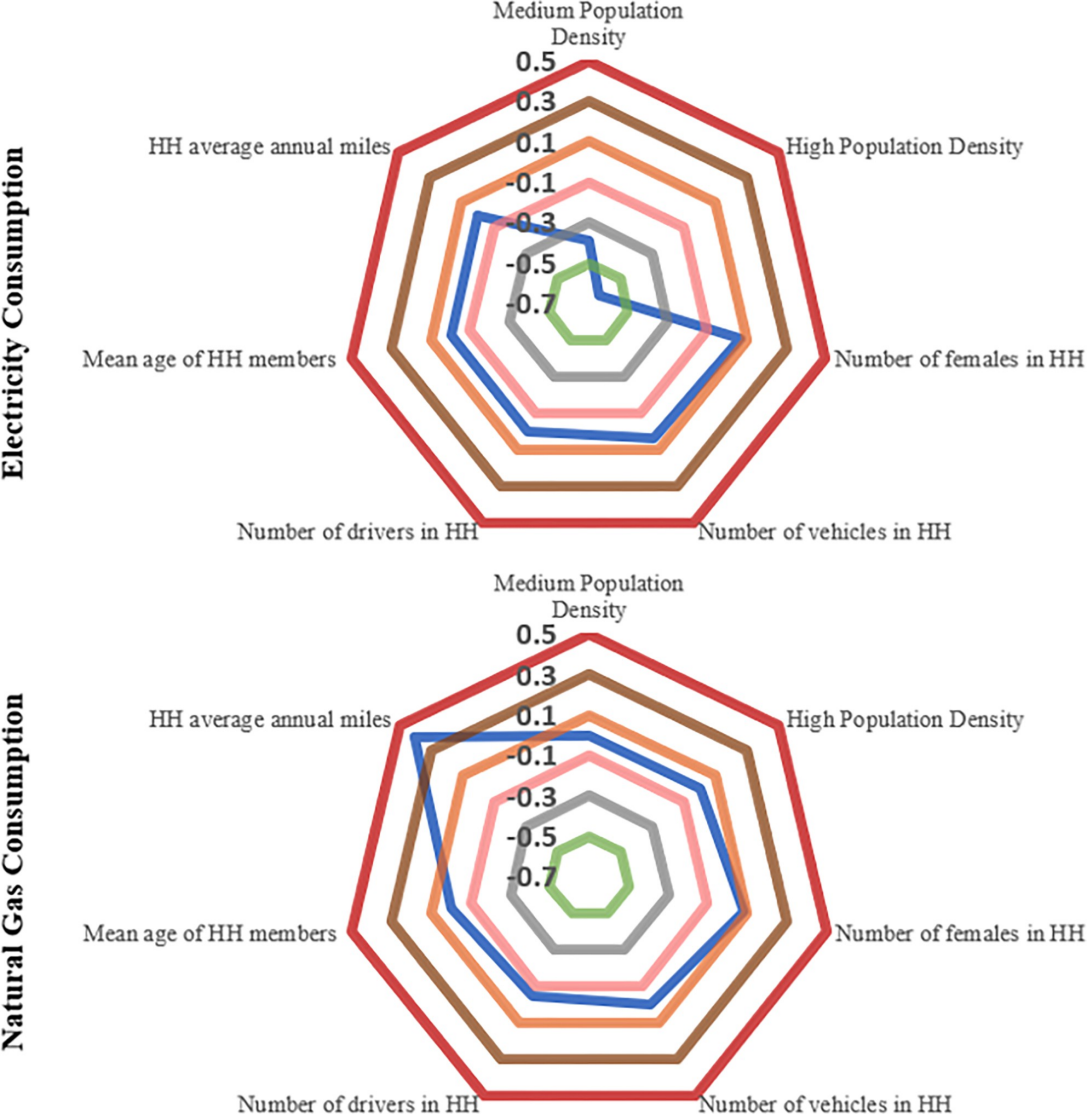
Graphical representation of NHTS variables’ impact on energy consumption.

In the fused dataset, several variables fused from NHTS are tested in our analysis. Fig 8 provides a quick mechanism for the reader to understand the impact of different NHTS related variables on energy consumptions.

The findings clearly highlight the reduced electricity usage in densely populated areas, perhaps indicative of the lower exposed floor area per capita [55]. In general, it appears that household with more females tend to use more electricity and natural gas relative to other households. This effect is perhaps the manifestation of the link between female and different activities in home including cooking, water heating, nurturing and cleaning [55]. Further, the estimated results show that the number of vehicles in a household is positively associated with household electricity consumption while a negative relationship is observed between the usage of natural gas and number of drivers in the household. The negative effect of the number of drivers in the household on its natural gas consumption may be attributed to the lesser time spent in houses as the ability to drive might encourage activities outside the home [56]. Interestingly, average age of a household (considering all members) reveals a negative effect on overall electricity consumption suggesting a reduced electricity use in a unit with older individuals. While this might seem counter intuitive on first glance as you would expect senior individuals to spend more time at home. However, the use of certain appliances such as deep freezer, dishwasher, tumble dryer and computers (and other devices) are relatively lower in houses with senior individuals and thus contribute to reduced electricity use [57,58]. Finally, average annual miles driven variable is found to be positively associated with natural gas consumption. This result is quite interesting and warrants further research. Overall, the findings are consistent with expectations and speak to the important role played by different factors in affecting residential energy demand.

#### 6.2.3 Weight component

As discussed earlier, variables used in the weight component are common variables present in both datasets that are not considered for matching. In terms of the electricity demand model, we find two variables: difference in household size and number of adults to exert significant impact on the weight component. The reader would note that a 0 difference means household from RECS and the fused household from NHTS has similar characteristics with respect to household size and number of adults. As expected, we find a negative impact for both of these variables on the electricity consumption model. The results indicates that the records having higher differences in household size and no. of adults will have lower weight contributions to the electricity consumption model. In the natural gas model, we observe a similar finding for “number of adults” variable difference. In the natural gas model, we also observe that contribution of a record is substantially higher when the ethnicity of the household matches with the fused household ethnicity.

### 6.3 Validation analysis

The model estimation results clearly illustrate the improved performance of the proposed model. In this section, we conduct a validation exercise, to evaluate the performance of the proposed LWLR model on the records not used for model estimation (hold-out sample). In the validation exercise, the performance of the fused LWLR model (with additional variables from NHTS and latent weight) is compared with the simple SLR model (employed with data form RECS only without fusing any record from the NHTS database) and equal weight EWLR model (with additional variables from NHTS and equal weight). The comparison exercise across the three models is conducted based on the predictive log-likelihood (LL) and BIC values.

The validation exercise is initially conducted with the 4000 record RECS estimation sample and 1686 record RECS validation sample. However, we realize that sample size in estimation could play a critical role in model performances [59] and hence we considered the influence of different sample sizes in model estimation by estimating the two model systems for different samples. Subsequently, to account for the impact of RECS sample size, we also conduct the validation exercise for different estimation and validation samples. In particular, from the RECS data, we randomly draw samples with 1,000; 2,000; 3,000; 4,000 and 5,000 households for estimation and for each estimation sample, the remaining households are considered as the hold-out samples. For example, RECS survey data provides information on 5,686 households. Out of these, for the first scenario, we considered 1,000 households as our estimation sample and the remaining 4,686 households are used for our validation exercise. For all these estimation and hold-out samples, we fused 15 records (K-15) from the NHTS dataset to the RECS dataset based on similar census division and location of the household. For the fused dataset, SLR, EWLR and LWLR models are estimated, and their performances based on predictive LL is compared. Further, as discussed earlier, for each record in the RECS data, there could be several potential matching records from the NHTS database and selecting 15 randomly out of these might introduce bias. Therefore, within each estimation and hold-out samples, we create 15 fused datasets (N), estimate (for estimation sample)/predict (for validation sample) the LL for each dataset across each model and finally compare the two models based on the average LL measures. The validation results are presented in Table 3.

**Table 3:** Model validation results.

Energy Source	Sample size	Avg. LL^*^ comparison for Estimation Sample					Avg. LL comparison for Validation Sample				
		*SLR*	*EWLR*	*LWLR*	*Improvement* *(EWLR~SLR)*	*Improvement* *(LWLR~EWLR)*	*SLR*	*EWLR*	*LWLR*.	*Improvement* *(EWLR~SLR)*	*Improvement* *(LWLR~EWLR)*
**Electricity**	Est.* 1000 Val** 4686	-766.69	-717.79	-708.68	97.80	18.22	-3566.73	-3398.23	-3351.86	337.00	92.73
	Est. 2000 Val. 3686	-1543.77	-1471.97	-1451.86	143.61	40.21	-2784.64	-2643.57	-2607.82	282.16	71.49
	Est. 3000 Val. 2686	-2274.54	-2147.40	-2120.08	254.29	54.62	-2048.79	-1954.14	-1921.53	189.31	65.22
	Est. 4000 Val. 1686	-2997.01	-2814.00	-2776.67	366.02	74.66	-1288.75	-1245.62	-1233.79	86.26	23.67
	Est. 5000 Val. 686	-3805.91	-3609.82	-3557.94	392.19	103.76	-511.79	-481.86	-472.56	59.86	18.61
**Natural Gas**	Est. 1000 Val. 4686	-1232.66	-1203.77	-1202.05	57.78	3.45	-5716.43	-5534.19	-5527.69	364.48	13.01
	Est. 2000 Val. 3686	-2358.39	-2305.03	-2300.19	106.72	9.69	-4584.61	-4407.48	-4402.18	354.26	10.59
	Est. 3000 Val. 2686	-3557.13	-3444.75	-3437.98	224.78	13.53	-3381.99	-3283.52	-3280.42	196.95	6.19
	Est. 4000 Val. 1686	-4891.95	-4722.44	-4712.96	339.01	18.98	-2035.04	-1945.81	-1943.74	178.48	4.14
	Est. 5000 Val. 686	-6086.97	-5881.82	-5871.24	410.30	21.16	-837.01	-829.00	-827.29	16.03	3.41

Note: Est* = Estimation sample size.

Val** = Validation sample size.

Table 3 presents the validation results for two energy use for electricity and natural gas. For each sample size, the average log-likelihood over N = 15 samples for SLR, EWLR, LWLR model and the improvement (computed as 2*(LL _EWLR_- LL_SLR_) and 2*(LL _LWLR_- LL_EWLR_) are presented. In all cases, the LWLR model shows clear improvement. The improvement is consistent i.e. the improvement is higher as the dataset size increases for estimation and validation samples. We compare these improvements to the critical chi-square values for the models. For electricity EWLR model, we have 5 additional variables compared to SLR model providing a critical 95% chi-square value of 11.070. The improvements values presented are clearly higher than the critical value. Further, the LWLR model with 2 additional variables outperformed the EWLR model as indicated by the higher log-likelihood ratio value relative to the corresponding critical chi-square value (5.991 for 2 variables). Similar findings are also observed in the natural gas model. The EWLR model (3 additional variables from SLR model for natural gas) improvement for all the samples are also well over the critical chi-square value. The LWLR model provides superior performance for majority of the samples (7 out of 10 samples) compared to the EWLR model in predicting the natural gas consumption. So, from the results, we can conclude that model improvement via fusion and latent weight is consistent across estimation and validation samples. The validation results clearly highlight how new variables from the NHTS dataset contribute to improvement in predicting energy consumption. In summary, the results clearly provide evidence that the proposed algorithm offers enhanced explanatory power and predictive capability. The reader would note the adoption of other metrics such as BIC offer similar results and are not included for the sake of brevity.

## 7 Conclusion

The current research is geared towards proposing and testing the efficacy of a simple yet statistically valid fusion approach to link the information from two disparate datasets into a unified database. In particular, the current approach augments RECS (source) data with additional variables from NHTS (donor) dataset with a focus on improving the quality of the energy model (two energy sources are considered: electricity and natural gas). The NHTS dataset was considered to incorporate additional variables such as socio-demographics, vehicle ownership, household location and travel patterns that are not available in the RECS data. The effectiveness of the proposed fusion method is rigorously tested with a well-crafted experimental design evaluating the influence of multiple independent variables for matching and fusing, fusion sample sizes and weight functions.

The analysis involves a series of model estimations, starting with a model focusing solely on RECS data (unfused model, SLR) and extending to models considering fused datasets with equal (EWLR model) and probabilistic weight allocations (LWLR model). The model fit comparison exercise demonstrates a clear improvement in the performance of the fused models, thereby supporting our hypothesis that the fusion of RECS and NHTS datasets enhances the performance of the energy model. Notably, within the fused models, the probabilistic weighting approach outperforms the equal weight approach, underscoring the critical role of the weight function in further improving the energy model’s accuracy. To further illustrate the applicability of the proposed fusion algorithm, we conduct a validation exercise comparing the fused model with probabilistic weight allocation to its counterparts across different estimation and validation samples. The results consistently show that the LWLR model with probabilistic weighting approach maintains its superior performance regardless of sample size and variable of interest, reinforcing the robustness of the fusion methodology. In terms of findings, we found several variables from the NHTS dataset to significantly impact residential energy demand, which are absent in the RECS data. Specifically, energy consumption is likely to be higher in houses with higher number of female and vehicles while factors like population density, number of drivers in the house and average age of household members reveals a negative relationship with the overall energy consumption.

In summary, the behavioral fusion algorithm proposed in the paper is simple to implement and relies on federally compiled NHTS and RECS data. The findings of the study clearly highlight the significant benefits of fusing two distinct datasets, as it results in better model fit, improved prediction accuracy, and enhanced explanatory power. For instance, the shift towards electric vehicles and the increasing trend of working from home significantly impact energy consumption patterns. The NHTS dataset, with its information on vehicle ownership and time spent at home, allows the proposed approach to address these evolving trends effectively. Further, the proposed fusion algorithm can be applied across various sectors, such as energy use and transportation planning. One possible application could be to integrate household travel survey data with location-based smartphone data to enhance spatiotemporal coverage and improve demand analysis. Additionally, the algorithm can be used to develop short-term forecasting methods for energy use by combining smart energy sensor data with RECS and NHTS data, offering a more dynamic and continuous prediction framework.

The reader will note that the data fusion process can be time-intensive for large datasets. The overall fusion process relies on two important steps: what variables to use for matching and how many matches to consider. Now, for any two datasets, if we have p number of matching variables, the potential combinations of variables that need to be explored in the analysis is $2^{p}-1(pC_{1}+pC_{2}+\cdots pC_{p-1})$. After determining the best set of matching variables, the next step is to find the optimal number of fused records as including all possible matching records could result in an excessively large dataset, making the model computationally demanding to run. The reader would note that a higher number of matching records does not always contribute to an improvement in the model (as shown in our analysis). Therefore, it is essential to optimize both the matching variables and the number of fused records to achieve a balance between model accuracy and computational efficiency. While this process can be time-consuming, it is not computationally complex, especially with the advanced computational power available today. The same considerations apply to large datasets, where the methodology remains feasible due to the scalability of modern computational resources. Thus, the computational cost, although significant, is manageable and does not pose a major limitation to applying the proposed method to very large datasets.

## Abbreviations

ANN: Artificial Neural Network
BIC: Bayesian Information Criterion
BPNN: Back Propagation Neural Network
EIA: US Energy Information Administration
EV: Electric Vehicle
EWLR: Equal weight regression model
FAF: Freight Analysis Framework
FARs: Fatality Analysis Reporting System
FHWA: Federal Highway Administration
GES: Generalized Estimates System
GIS: Geographic Information System
GPS: Global Positioning System
HH: Household
HVAC: Heating, ventilation, and air conditioning
IOT: Internet of Things
KNN: K-Nearest Neighbour
LBS: Location Based Service
LDA: Latent Dirichlet Allocation
LL: Log-likelihood
LSTM: Long Short-Term Memory
LWLR: Latent Weight Regression Model
MDCEV: Multiple Discrete Continuous Extreme Value
MNL: Multinomial Logit
NHTS: National Household Travel Survey
RECS: Residential Energy Consumption Survey
SLR: Simple Linear Regression model
SVM: Support Vector Machine
TS: Transearch
US: United States
**Notation**: **Description**
i: Index for households in the RECS dataset
K: Number of possible matches from the NHTS dataset
d: Index for different energy sources (electricity, natural gas
y_d,i_: Observed log-normal of energy usage for household i and energy source d
Q_d,ik_: Predicted log-normal of energy usage for household i and the K^th^ fused record for energy source d
X_ik_: Vector of attributes from the source dataset influencing energy demand
S_ik_: Vector of attributes from the donor dataset affecting energy demand
β′: Coefficients corresponding to X_ik_
γ′: Coefficients corresponding to S_ik_
ε_ik_: Independently and identically distributed error term with zero mean and variance σ^2^
P(Q_ik_): the probability for HH i for the K^th^ fused records to have y_i_ energy demand
ϕ(.): Standard normal probability density function
P_ik_: matched weightage propensity
Z_ik_: Vector of attributes considered for matching
∝: Corresponding vector to be estimated for Z_ik_
Q_i_: weighted probability that HH i has y_i_ energy demand
LL: Log-likelihood function for the fused dataset energy demand

## References

[1] US-EIA2020. Frequently Asked Questions (FAQs)—U.S. Energy Information Administration (EIA), https://www.eia.gov/tools/faqs/faq.php?id=87&t=1 (accessed 11 November 2022).

[2] 2022 W. United States Population (2022)—Worldometer, https://www.worldometers.info/world-population/us-population/ (accessed 11 November 2022).

[3] US-DOE 2019. Energy Data Facts | Residential Program Solution Center, https://rpsc.energy.gov/energy-data-facts (accessed 26 July 2021).

[4] US-EIA 2019. U.S. Energy Information Administration—EIA—Independent Statistics and Analysis, https://www.eia.gov/totalenergy/data/browser/index.php?tbl=T02.01#/?f=A&start=2019&end=2020&charted=3-6-9-12 (accessed 10 May 2022).

[5] Bhowmik T, Tirtha SD, Iraganaboina NC, et al. A comprehensive analysis of COVID-19 transmission and mortality rates at the county level in the United States considering socio-demographics, health indicators, mobility trends and health care infrastructure attributes. *PLoS One*; 16. Epub ahead of print 2021. doi: 10.1371/journal.pone.0249133 33793611 PMC8016225

[6] US-EIA2021. Total Energy Monthly Data—U.S. Energy Information Administration (EIA), https://www.eia.gov/totalenergy/data/monthly/ (accessed 10 May 2022).

[7] Electrek. Global market share of electric cars more than doubled in 2021 as the EV revolution gains steam—Electrek, https://electrek.co/2022/02/02/global-market-share-of-electric-cars-more-than-doubled-2021/ (accessed 10 May 2022).

[8] Kapustin NO, Grushevenko DA. Long-term electric vehicles outlook and their potential impact on electric grid. *Energy Policy* 2020; 137: 111103.

[9] Data Fusion—an overview | ScienceDirect Topics, https://www.sciencedirect.com/topics/computer-science/data-fusion (accessed 24 July 2021).

[10] Varlamis I, Sardianos C, Chronis C, et al. Smart fusion of sensor data and human feedback for personalized energy-saving recommendations. *Appl Energy* 2022; 305: 117775.

[11] Wang Z, Hong T, Piette MA. Data fusion in predicting internal heat gains for office buildings through a deep learning approach. *Appl Energy* 2019; 240: 386–398.

[12] Guarino F, Croce D, Tinnirello I, et al. Data fusion analysis applied to different climate change models: An application to the energy consumptions of a building office. *Energy Build* 2019; 196: 240–254.

[13] Gouveia JP. Understanding electricity consumption patterns in households through data fusion of smart meters and door-to-door surveys. *Eceee 2015* 2015; 957–966.

[14] Himeur Y, Alsalemi A, Al-Kababji A, et al. Data fusion strategies for energy efficiency in buildings: Overview, challenges and novel orientations. *Inf Fusion* 2020; 64: 99–120.

[15] Yang C, Zhang Y, Zhan X, et al. Fusing Mobile Phone and Travel Survey Data to Model Urban Activity Dynamics. *J Adv Transp*; 2020. Epub ahead of print 2020. doi: 10.1155/2020/5321385

[16] Montero L, Ros-Roca X, Herranz R, et al. Fusing mobile phone data with other data sources to generate input OD matrices for transport models. *Transp Res Procedia* 2019; 37: 417–424.

[17] Sivakumar A, Polak J. An exploration of data pooling techniques: Modelling activity participation and household technology holdings Abstract: 7228.

[18] Liao C-F. Fusing Public and Private Truck Data to Support Regional Freight Planning and Modeling Traffic Information for People with Vision Impairment View project Fusing Public and Private Truck Data to Support Regional Freight Planning and Modeling, https://www.researchgate.net/publication/229038251 (2016, accessed 25 July 2021).

[19] Momtaz SU, Eluru N, Anowar S, et al. Fusing Freight Analysis Framework and Transearch Data: Econometric Data Fusion Approach with Application to Florida. *J Transp Eng Part A Syst* 2020; 146: 04019070.

[20] Zhao D, Balusu SK, Sheela PV, et al. Weight-categorized truck flow estimation: A data-fusion approach and a Florida case study. *Transp Res Part E Logist Transp Rev* 2020; 136: 101890.

[21] Martín Y, Cutter SL, Li Z. Bridging Twitter and Survey Data for Evacuation Assessment of Hurricane Matthew and Hurricane Irma. Epub ahead of print 2020. doi: 10.1061/(ASCE)NH.1527-6996.0000354

[22] Yasmin S, Eluru N, Pinjari AR. Pooling data from fatality analysis reporting system (FARS) and generalized estimates system (GES) to explore the continuum of injury severity spectrum. *Accid Anal Prev* 2015; 84: 112–127. doi: 10.1016/j.aap.2015.08.009 26342892

[23] Jiang S, Ferreira J, Gonzalez MC. Activity-Based Human Mobility Patterns Inferred from Mobile Phone Data: A Case Study of Singapore. *IEEE Trans Big Data* 2016; 3: 208–219.

[24] Xu Y, Shaw SL, Zhao Z, et al. Understanding aggregate human mobility patterns using passive mobile phone location data: a home-based approach. *Transportation (Amst)* 2015; 42: 625–646.

[25] Bedir M, Hasselaar E, Itard L. Determinants of electricity consumption in Dutch dwellings. *Energy Build* 2013; 58: 194–207.

[26] Huang WH. The determinants of household electricity consumption in Taiwan: Evidence from quantile regression. *Energy* 2015; 87: 120–133.

[27] Belaïd F, Garcia T. Understanding the spectrum of residential energy-saving behaviours: French evidence using disaggregated data. *Energy Econ* 2016; 57: 204–214.

[28] Wiesmann D, Lima Azevedo I, Ferrão P, et al. Residential electricity consumption in Portugal: Findings from top-down and bottom-up models. *Energy Policy* 2011; 39: 2772–2779.

[29] Dale L, Fujita S, Vasquez F, et al. Price Impact on the Demand for Water and Energy in California Residences. *Public Interes Energy Res Progr Reports CEC-500-2009-032-D*, *Calif Energy Comm Sacramento*, *CA*, http://www.energy.ca.gov/2009publications/CEC-500-2009-032/CEC-500-2009-032-F.PDF (2009, accessed 25 July 2021).

[30] Mansur ET, Mendelsohn R, Morrison W. Climate change adaptation: A study of fuel choice and consumption in the US energy sector. *J Environ Econ Manage* 2008; 55: 175–193.

[31] Iraganaboina NC, Eluru N. An examination of factors affecting residential energy consumption using a multiple discrete continuous approach. *Energy Build*; 240. Epub ahead of print 2021. doi: 10.1016/j.enbuild.2021.110934

[32] Pinjari AR, Bhat C. Computationally efficient forecasting procedures for Kuhn-Tucker consumer demand model systems: Application to residential energy consumption analysis. *J Choice Model*; 39. Epub ahead of print 2021. doi: 10.1016/j.jocm.2021.100283

[33] Sailor DJ, Muñoz JR. Sensitivity of electricity and natural gas consumption to climate in the U.S.A.—Methodology and results for eight states. *Energy* 1997; 22: 987–998.

[34] Dubin JA, McFadden DL. An Econometric Analysis of Residential Electric Appliance Holdings and Consumption. *Econometrica* 1984; 52: 345.

[35] Harold J, Lyons S, Cullinan J. The determinants of residential gas demand in Ireland. *Energy Econ* 2015; 51: 475–483.

[36] Anderson B, Lin S, Newing A, et al. Electricity consumption and household characteristics: Implications for census-taking in a smart metered future. *Comput Environ Urban Syst* 2017; 63: 58–67.

[37] Nesbakken R. Energy consumption for space heating: A discrete-continuous approach. *Scand J Econ* 2001; 103: 165–184.

[38] Boomsma C, Jones R V, Pahl S, et al. Energy Saving Behaviours Among Social Housing Tenants: Exploring the Relationship With Dwelling Characteristics, Monetary Concerns, and Psychological Motivations. *4th Eur Conf Behav Energy Effic (Behave 2016)* 2016; 8–9.

[39] Wu C, Thai J, Yadlowsky S, et al. Cellpath: Fusion of Cellular and Traffic Sensor Data for Route Flow Estimation via Convex Optimization. *Transp Res Procedia* 2015; 7: 212–232.

[40] Iqbal MS, Choudhury CF, Wang P, et al. Development of origin-destination matrices using mobile phone call data. *Transp Res Part C Emerg Technol* 2014; 40: 63–74.

[41] Li X, Wen J. System identification and data fusion for on-line adaptive energy forecasting in virtual and real commercial buildings. *Energy Build* 2016; 129: 227–237.

[42] Jiang L, Wang X, Li W, et al. Hybrid Multitask Multi-Information Fusion Deep Learning for Household Short-Term Load Forecasting. *IEEE Trans Smart Grid* 2021; 12: 5362–5372.

[43] Tan SY, Jacoby M, Saha H, et al. Multimodal sensor fusion framework for residential building occupancy detection. *Energy Build* 2022; 258: 111828.

[44] Xie J, Zhong Y, Xiao T, et al. A multi-information fusion model for short term load forecasting of an architectural complex considering spatio-temporal characteristics. *Energy Build* 2022; 277: 112566.

[45] Peng D, Zhao J, Xu T. Intelligent Building Data Fusion Algorithm by Using the Internet of Things Technology. *J Phys Conf Ser* 2021; 2143: 012030.

[46] Fawzy D, Moussa S, Badr N. The spatiotemporal data fusion (Stdf) approach: Iot-based data fusion using big data analytics. *Sensors* 2021; 21: 7035. doi: 10.3390/s21217035 34770342 PMC8588564

[47] Wang W, Chen J, Hong T. Occupancy prediction through machine learning and data fusion of environmental sensing and Wi-Fi sensing in buildings. *Autom Constr* 2018; 94: 233–243.

[48] Nesa N, Banerjee I. IoT-Based Sensor Data Fusion for Occupancy Sensing Using Dempster-Shafer Evidence Theory for Smart Buildings. *IEEE Internet Things J* 2017; 4: 1563–1570.

[49] He N, Liu L, Qian C, et al. Air Conditioning Load Prediction Based on Data Fusion Model. *SSRN Electron J*. Epub ahead of print 18 March 2022. doi: 10.2139/ssrn.4059927

[50] De Silva D, Alahakoon D, Yu X. A data fusion technique for smart home energy management and analysis. *IECON Proc (Industrial Electron Conf* 2016; 4594–4600.

[51] Wijayasekara D, Manic M. Data-fusion for increasing temporal resolution of building energy management system data. *IECON 2015 - 41st Annu Conf IEEE Ind Electron Soc* 2015; 4550–4555.

[52] CBC News. Why mobile home residents are paying for more electricity | CBC News, https://www.cbc.ca/news/canada/british-columbia/mobile-home-bc-hydro-report-1.5861458 (accessed 26 July 2021).

[53] Reyna JL, Chester M V. Energy efficiency to reduce residential electricity and natural gas use under climate change. *Nat Commun* 2017; 8: 1–12. 28504255 10.1038/ncomms14916 PMC5440627

[54] 2020 R. All-Electric New Homes: A Win for the Climate and the Economy—RMI, https://rmi.org/all-electric-new-homes-a-win-for-the-climate-and-the-economy/ (accessed 24 May 2022).

[55] USAToday. Sustainability study shows that women consume more energy, https://www.utsa.edu/today/2015/05/afamia.html (accessed 27 July 2021).

[56] Golob TF, Brownstone D. The Impact of Residential Density on Vehicle Usage and Energy Consumption. *J Urban Econ* 2009; 65: 91–98.

[57] Brounen D, Kok N, Quigley JM. Residential energy use and conservation: Economics and demographics. *Eur Econ Rev* 2012; 56: 931–945.

[58] Leahy E, Lyons Sean S. Energy use and appliance ownership in Ireland. *Energy Policy* 2010; 38: 4265–4279.

[59] Bhowmik T, Yasmin S, Eluru N. A New Econometric Approach for Modeling Several Count Variables: A Case Study of Crash Frequency Analysis by Crash Type and Severity. *Transp Res Part B Methodol* 2021; 153: 172–203.

